# Causal risk and protective factors for rheumatoid arthritis: a comprehensive mendelian randomization systematic review and meta-analysis

**DOI:** 10.3389/fgene.2026.1774582

**Published:** 2026-03-19

**Authors:** Xu Han, Zhenzhen Han, Siyu Wang, Yafei Zhang, Ying Liu, Jing Zhang, Jinlong Yu, Danyang Cui, Xiaoxia Ma, Jiazhu Jin, Lei Zhang, Hongtao Guo, Miao Jiang

**Affiliations:** 1 Institute of Basic Research in Clinical Medicine, China Academy of Chinese Medical Sciences, Beijing, China; 2 Department of Rheumatology, The First Affiliated Hospital of Henan University of CM, Zhengzhou, Henan, China; 3 National Data Center of Traditional Chinese Medicine, China Academy of Chinese Medical Sciences, Beijing, China; 4 General Hospital of Northern Theater Command, Shenyang, Liaoning, China

**Keywords:** mendelian randomization, protective factor, rheumatoid arthritis, risk factor, systematic review and meta-analysis

## Abstract

**Objective:**

To systematically synthesize Mendelian randomization (MR) evidence exploring causal associations between various exposures and rheumatoid arthritis (RA).

**Methods:**

Systematic searches were conducted to identify eligible two-sample MR studies evaluating causal links between exposures and RA. Data extraction encompassed exposure types, genetic instruments, and analytical methods. The quality of evidence was evaluated based on STROBE-MR guidelines and evidence strength grades. Meta-analyses were performed using random-effects model with restricted maximum likelihood estimation and Hartung-Knapp-Sidik-Jonkman confidence intervals to summarize causal estimates.

**Results:**

A total of 117 studies were included, comprising 496 MR associations across 248 unique exposures. Of these, 200 exposure-RA associations across 65 unique exposures were eligible for meta-analysis. The primary analysis identified smoking behavior (OR = 1.39, 95% CI 1.30–1.49) and hypothyroidism (OR = 1.55, 1.34–1.81) as significant risk factors with high-quality evidence. While television viewing (OR = 2.27, 1.77–2.90) and cholangitis (OR = 1.14, 1.06–1.23) showed nominal positive associations. Conversely, cognitive function (OR = 0.74, 0.68–0.81) and Interleukins-1 receptor antagonist (OR = 0.82, 95% CI 0.73–0.92) were identified as a nominally protective factor. In subtype analyses, basal metabolic rate emerged as a potential shared risk factor for both seropositive and seronegative RA. Overall, 7.66% and 26.01% of all associations were classified as providing Robust (I) and Probable (II) evidence, respectively.

**Conclusion:**

This comprehensive review clarifies the causal landscape of RA by distinguishing between significant causal drivers and potential associations. The findings highlight smoking cessation and thyroid function management as critical, evidence-based targets for RA prevention. However, the high proportion of low-quality evidence underscores the need for future high-quality MR studies to validate weaker signals.

**Systematic Review Registration:**

https://www.crd.york.ac.uk/PROSPERO/view/CRD42024573056, identifier CRD42024573056.

## 1 Introduction

Rheumatoid Arthritis (RA) is a chronic autoimmune disorder that primarily targets the synovial joints, leading to persistent inflammation, symmetrical arthritis, joint pain, swelling, and progressive joint dysfunction. In severe cases, RA can result in significant joint deformity and permanent disability (Conforti et al., 2021; Cojocaru et al., 2010). According to the Global Burden of Disease Study 2019, approximately 17.6 million people worldwide were living with RA in 2020, with an age-standardized prevalence rate of 208.8 cases per 100,000 population, which 14.1% increase since 1990 (GBD, 2023). Current models predict that the global prevalence of RA will continue to rise through 2050, underscoring the urgent need for effective management strategies (GBD, 2023). The economic burden of RA is considerable; for instance, in the United Kingdom alone, the annual cost associated with RA—including disability and sick leave—was estimated to range between £380 million and £4.8 billion, with the combined annual cost of RA and osteoarthritis-induced sick leave and job loss reaching an estimated £100 billion in 2019 (Perera et al., 2024). Additionally, the disease often compels about one-third of patients to abandon their careers within 2 years of disease onset, further exacerbating the socio-economic impact (Perera et al., 2024). There is currently no cure for RA, which makes its early detection and prevention all the more critical.

RA is recognized as a multifactorial disease influenced by both genetic predispositions and environmental exposures (Bergstedt et al., 2022), yet the etiology of RA remains incompletely understood. Traditional epidemiologic studies have identified a variety of risk factors associated with RA, including smoking and obesity (Deane et al., 2017; Zaccardelli et al., 2019; Liu et al., 2019a; Tedeschi et al., 2017), gender (Alpizar-Rodriguez et al., 2017; Alpízar-Rodríguez et al., 2017) and certain genetic markers (Karlson et al., 2010). However, it remains challenging to establish clear causal relationships between these risk factors and RA. Previous classic observational studies are often plagued by potential confounding variables and reverse causation, which can obscure true causal pathways and lead to misleading conclusions.

Mendelian randomization (MR) has emerged as a robust methodological approach to address these limitations. By utilizing genetic variants as instrumental variables (IVs), MR allows for the estimation of the causal effect of modifiable exposures on disease outcomes, thereby mitigating biases related to confounding and reverse causation commonly encountered in traditional observational studies (Lawlor et al., 2008). The increasing application of MR in epidemiological research, particularly in the study of RA, underscores its potential to elucidate the underlying causal mechanisms and identify actionable risk factors.

To date, systematic reviews and meta-analyses focusing on the impact of specific exposures—such as smoking, alcohol consumption, coffee intake, and physical activity—on RA using MR approaches have been conducted (Sun et al., 2021a; Wang et al., 2023). However, these reviews addressed only a subset of potential risk factors, thus there remains a need for a comprehensive systematic review and meta-analysis that synthesizes the full spectrum of MR studies investigating a wide array of exposures related to RA. An exhaustive evaluation would allow a more holistic understanding of the etiological factors contributing to RA and identify novel targets for intervention and prevention.

Therefore, the objective of this study is to systematically identify, evaluate, and synthesize evidence from MR studies on various modifiable and non-modifiable risk factors associated with RA, so as to improve our understanding of RA etiology, point out the inconsistencies in the existing literature, and highlight potential therapeutic intervention.

## 2 Methods

This systematic review was reported in accordance with the Preferred Items for Systematic Reviews and Meta-Analysis (PRISMA) guidelines (Page et al., 2021). The corresponding protocol for the systematic review has been registered in the International Prospective Register of Systematic Reviews (PROSPERO CRD42024573056, and the publicly accessible website is https://www.crd.york.ac.uk/PROSPERO/view/CRD42024573056).

### 2.1 Search strategy

A comprehensive literature search was conducted across multiple databases including PubMed, Embase, and Web of science. The databases were searched for relevant citations published from their inception to 1 January 2026 with keywords related to RA and MR (Supplementary Table S1). Reference lists of selected articles were also reviewed to identify additional relevant studies.

### 2.2 Eligibility criteria

Studies were included if they meet the following criteria: 1) MR analysis without restrictions on sample size; 2) Reported the impact of factors for RA; 3) Two Sample MR. We exclude editorials, letters to the editor, review articles, conference coverage, systematic reviews, case reports, and experimental animal studies. Repeatedly published studies were also excluded.

### 2.3 Literature screening

The article selection process was conducted by two independent reviewers. Disagreements between the reviewers regarding the included studies were resolved by discussion. The third reviewer made the decision if there was no reconciliation between the two reviewers. After excluding duplicated studies, studies were excluded which did not meet inclusion criteria by screening titles and abstracts. Then, the authors assessed full-text articles.

### 2.4 Data extraction

Data extraction was conducted independently by two authors using a standardized template. For each identified MR association, the following information was systematically recorded: 1) first author and publication year; 2) the specific exposure and its categorization; 3) the source, sample size, and population ancestry for both exposure and outcome GWAS summary statistics; 4) the number of IVs used; and 5) the primary reported effect estimate (Odds Ratio [OR] with its 95% Confidence Interval [CI]) and corresponding P-value. Any discrepancies encountered during the extraction process were resolved through discussion and, if necessary, adjudication by a third author. The estimate from the random-effects model was prioritized for extraction to conservatively account for potential heterogeneity, when multiple estimates were reported (e.g., fixed-effect and random-effects). To ensure data independence and avoid duplicate weighting, we rigorously verified sample overlap across the following dimensions: GWAS cohort/consortium name, sample size, GWAS ID, ancestry, and data release version. When multiple studies reported on the same exposure-outcome pair using overlapping datasets, we retained only the analysis with the most recent data version. Whereas distinct exposure phenotypes derived from the same population were treated as independent and included.

### 2.5 Risk of bias quality assessment

The risk of bias for each included study was assessed using a modified checklist derived from the STROBE-MR guidelines (Skrivankova et al., 2021). Based on the resulting scores, studies were categorized as high (>85%), moderate (75%–85%), or poor (<75%) quality (Tian et al., 2024; Davies et al., 2018; Boef et al., 2015). Subsequently, the overall strength of evidence for each exposure-outcome association was graded by adapting the framework proposed by Markozannes et al. (Markozannes et al., 2022). This framework classifies evidence as Robust (I), Probable (II), Suggestive (III), or Insufficient (IV), based on the statistical significance (P < 0.05) and directional consistency of effects across the primary and sensitivity analyses. Associations lacking sensitivity analyses were deemed Non-evaluable. This assessment was performed independently by two authors, with any disagreements adjudicated by a third.

### 2.6 Data synthesis

To address potential between-study heterogeneity and the small number of studies (k) typical in MR meta-analyses, a random-effects model was pre-specified as the primary analytical approach. The restricted maximum likelihood (REML) estimator was used to estimate the between-study variance (tau^2^). To prevent the high false-positive rates often associated with standard Wald-type confidence intervals (CIs) when k is small, the Hartung-Knapp-Sidik-Jonkman (HKSJ) method was employed to calculate 95% CIs and P-values for the pooled estimates. Given the large number of exposure-outcome associations tested, the Benjamini-Hochberg procedure was applied to control the False Discovery Rate (FDR). Associations with an FDR-corrected P-value (P_FDR) < 0.05 were considered significant association. Associations with a nominal P-value (P_HKSJ) < 0.05 but P_FDR ≥0.05 were considered nominally significant association.

Heterogeneity was quantified using Cochran’s Q test and the I^2^ statistic. Sensitivity analyses were conducted to verify the robustness of the primary findings: (1) using a fixed-effect model; (2) using a random-effects model with standard Wald-type CIs; and (3) performing leave-one-out analyses for associations with k ≥ 3. For exposures with k ≥ 10, publication bias was assessed using Egger’s test, Begg’s test, and the trim-and-fill method, and sources of heterogeneity were explored *via* subgroup analysis and meta-regression. All statistical analyses were performed using R Software.

## 3 Results

### 3.1 Literature search and selection

**FIGURE 1:**
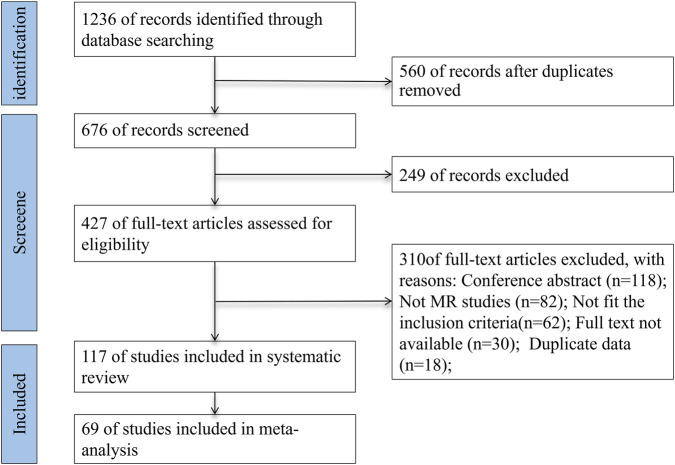
PRISMA flow diagram depicting the study selection process.

A comprehensive literature search yielded a total of 1,236 articles. After the removal of 560 duplicate records, 676 unique studies remained for further evaluation. Initial screening based on titles and abstracts led to the exclusion of 249 articles, resulting in 427 studies eligible for full-text review. Upon detailed examination, 310 studies were excluded for the following reasons: 118 were conference abstracts, 82 were non- MR studies, 62 did not meet the inclusion criteria, 30 lacked accessible full texts, and 18 involved duplicate data. Consequently, 117 and 69 studies were included in the final systematic review and meta-analysis, respectively (Figure 1).

### 3.2 Study characteristics

**FIGURE 2:**
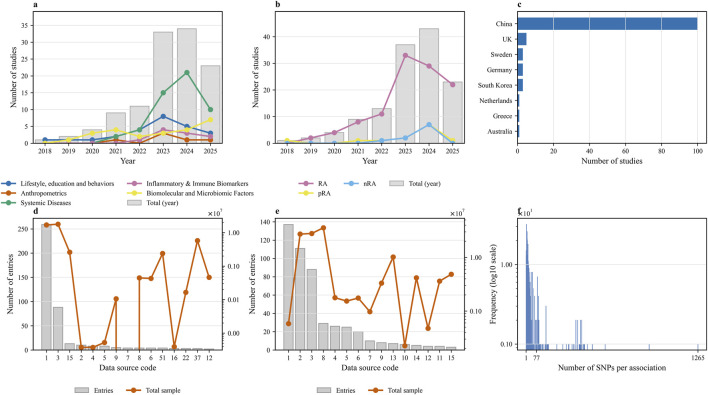
Descriptive characteristics of the included Mendelian randomization studies. **(a)** Annual publication trends of studies, stratified by major exposure categories. **(b)** Annual publication trends of studies, stratified by rheumatoid arthritis (RA) outcome subtypes (RA, seropositive RA [pRA], and seronegative RA [nRA]). **(c)** Geographical distribution of studies based on the country of the first or corresponding author’s institution. **(d)** Distribution of data sources for exposures, displaying the number of entries (grey bars) and the total sample size (orange line) for each source code. **(e)** Distribution of data sources for outcomes. **(f)** Frequency distribution of the number of single nucleotide polymorphisms (SNPs) used per association, with the frequency presented on a log10 scale. The numerical codes on the x-axes of panels **(d,e)** correspond to the full data source names listed in Supplementary Table S3.

A total of 117 publications were included in this review, providing 496 MR associations between 248 unique exposures and RA (Sun et al., 2021a; Wang et al., 2023), (Zhou et al., 2024; Zhou et al., 2023; Zhou et al., 2021; Zhao et al., 2023; Zhao et al., 2022; Zhang et al., 2024; Yuan et al., 2024; Yuan et al., 2022; Yuan and Larsson, 2020; Yu et al., 2021; Yu XH. et al., 2022; Yu X. et al., 2022; Yu et al., 2024; You et al., 2023; Yin et al., 2022; Ye et al., 2021; Yao et al., 2024; Yao et al., 2023; Yang S. et al., 2024; Yang et al., 2023a; Yang et al., 2023b; Yang M. et al., 2024; Yang J. et al., 2024; Yang H. et al., 2023; Yan et al., 2025; Xu et al., 2021; Xiang et al., 2023; Wu et al., 2024; Wu et al., 2023), (Wu et al., 2021; Wen et al., 2024; Wei et al., 2023; Wang et al., 2024; Teng et al., 2024; Tang et al., 2023; Tang et al., 2024; Tang et al., 2021; Syed et al., 2020; Sun et al., 2021b; Sun et al., 2023; Qu et al., 2020; Qiu et al., 2024; Qian et al., 2020; Pu et al., 2022; Peng et al., 2023; Pan et al., 2024; Meisinger et al., 2022; Ma et al., 2024; Lu and Huang, 2023; Liu Z. et al., 2024; Liu Q. et al., 2024; Liang et al., 2024; Li Y. et al., 2023; Li Y. et al., 2024; Li X. et al., 2023; Li T. et al., 2024; Li M. et al., 2024; Li K. et al., 2024; Li H. et al., 2023), (Li GS. et al., 2024; Li G. et al., 2024; Leavy et al., 2024; Larsson and Burgess, 2022; Lai et al., 2023; Kurilshikov et al., 2021; Karlsson et al., 2023; Jiang et al., 2021; Huang J. et al., 2023; Huang, 2023; Huang et al., 2023b; Huang et al., 2023c; Hu et al., 2024; Hong et al., 2024; Guo et al., 2022; Gu et al., 2023; Gao et al., 2023; Gao et al., 2022; Freuer and Meisinger, 2023; Fang et al., 2023; Fan et al., 2023; Duan et al., 2023; Du et al., 2024; Du et al., 2023; Dong et al., 2024; Cheng et al., 2024; Cheng et al., 2019; Chen et al., 2024; Chen et al., 2023; Chen and He, 2024), (Cao et al., 2023; Bae and Lee, 2019; Bae and Lee, 2018; Yang H. et al., 2025; Wu et al., 2025; Huang et al., 2026; Guo et al., 2025; Fan R. et al., 2025; Fan H. et al., 2025; Dai et al., 2025; Yi et al., 2025; Yang W. et al., 2025; Yang C. et al., 2025; Xiao S. et al., 2025; Xiao P. et al., 2025; Wei et al., 2025; Tian et al., 2025; Song et al., 2025; Ma et al., 2025; Luo et al., 2025; Lu et al., 2025; Li J. et al., 2025; Li H. et al., 2025; Lazanas et al., 2025; Huang W. et al., 2025; Huang J. et al., 2025). The included studies were published between 2018 and 2025, with a marked acceleration in publication volume observed from 2022 onwards (Figures 2a,b). Geographically, a significant majority of the studies originated from institutions in China (n = 100), followed distantly by the United Kingdom (n = 5), Sweden (n = 3), South Korea (n = 3), Germany (n = 3), Netherlands (n = 1), Australia (n = 1) and Greece (n = 1) (Figure 2c). The 248 unique exposures were classified into five main categories, with systemic diseases showing the most substantial increase as a research focus in recent years (Figure 2a). While most studies investigated RA as a general outcome, a smaller but growing number focused specifically on seropositive (pRA) or seronegative (nRA) subtypes (Figure 2b). The exposure data were predominantly derived from populations of European ancestry (431/496 associations). Analysis of the data sources for both exposures and outcomes revealed a reliance on a limited number of large-scale GWAS consortia, which were used repeatedly across studies (Figures 2d,e; Supplementary Table S3). Summary-level data for outcomes were primarily sourced from two large-scale GWAS consortia: the Okada et al. dataset (Okada et al., 2014), which defined RA cases based on clinical criteria, and the FinnGen consortium, where cases were identified using ICD codes. A comprehensive overview of these characteristics is presented in Figure 2, with detailed information for each study available in Supplementary Table S2.

### 3.3 Methodological approaches of included studies

The predominant MR methods employed across these analyses included Inverse Variance Weighting (IVW), MR-Egger regression, MR-PRESSO, Simple Mode, and Weighted Median approaches. Specifically, all 496 associations analyses utilized the IVW method. In addition, 371 analyses incorporated MR-Egger regression, 67 utilized MR-PRESSO, 215 employed the Simple Mode, and 382 applied the Weighted Median method. To enhance the robustness of the causal inferences, multiple MR methods were often employed concurrently. Among the analyses, 256 exposures were assessed using four or more MR methods, 137 used three methods, nine employed two methods, and 64 analyses relied solely on the IVW method. Regarding the genetic instruments, the number of single nucleotide polymorphisms (SNPs) utilized per association showed a highly right-skewed distribution, with a median of 14 SNPs (IQR: 7–38), indicating that most associations were constructed using a relatively small number of SNPs (Figure 2f).

### 3.4 Quality of included studies and strength of evidence

**FIGURE 3:**
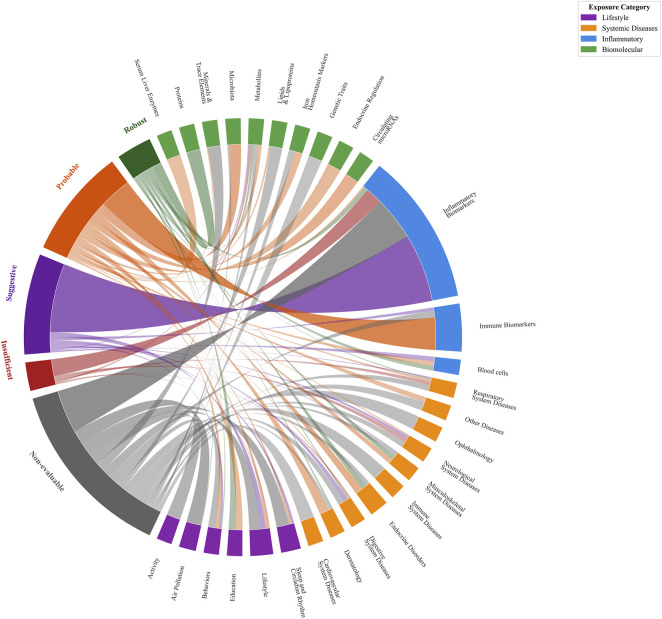
Distribution of evidence category for MR analyses by exposure categories.

The reporting quality of the 117 included studies was evaluated using a modified 14-point STROBE-MR checklist. The overall quality was high, with assessment scores ranging from 86% to 100%. The detailed STROBE-MR assessment for each study is provided in Supplementary Table S4. Subsequently, the evidentiary strength of the 496 MR associations was graded based on the consistency and significance of the findings. Overall, 38 associations (7.66%) presented “Robust” evidence, 129 (26.01%) were “Probable,” and 107 (21.57%) were “Suggestive.” A smaller portion was classified as “Insufficient” (30 associations, 6.05%), while a significant number were deemed “Non-evaluable” (192 associations, 38.71%). A detailed distribution of these evidence grades is presented in Figure 3; Supplementary Table S5.

### 3.5 Descriptive analysis

**FIGURE 4:**
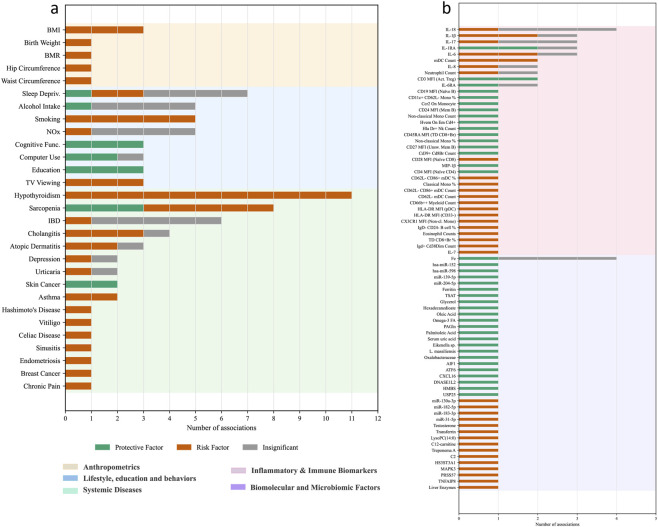
Descriptive synthesis of the evidence landscape from MR studies on exposures associated with RA. **(a)** Non-molecular exposures associated with RA in the included MR studies. **(b)** Molecular and circulating biomarker exposures associated with RA in the included MR studies. Note: The number indicates the count of individual MR studies that reported the corresponding outcome. Abbreviation: BMI, Body Mass Index; BMR, Basal Metabolic Rate; Sleep Depriv., Sleep Deprivation; NOx, Nitrogen Oxides; Cognitive Func., Cognitive Function; TV Viewing, Television Viewing; IBD, Inflammatory bowel disease; IL-18, Interleukin-18; IL-1β, Interleukin-1b; IL-17, Interleukin-17; IL-1RA, Interleukin-1 Receptor Antagonist; IL-6, Interleukin-6; mDC, Myeloid Dendritic Cells; IL-8, Interleukin-8; CD3 MFI (Act. Treg), Cd3 On Cd39+ Activated Treg; IL-6RA, Interleukin-6 Receptor Antagonist; Mono, Monocyte; MIP-1β, Macrophage Inflammatory Protein-1B; IL-7, Interleukin-7; Omega-3 FA, Omega-3 Fatty Acids; Eikenella sp.,Eikenella sp001648475; AIF1, Allograft inflammatory factor 1; ATF6, Cyclic AMP-dependent transcription factor ATF-6 alpha; CXCL16, C-X-C motif chemokine 16; DNASE1L2, Deoxyribonuclease-1-like 2; HMBS, Porphobilinogen deaminase; USP25, Ubiquitin carboxyl-terminal hydrolase 2; HS3ST3A1, Heparan sulfate glucosamine 3-O-sulfotransferase 3A1; MAPK3, MAP kinase-activated protein kinase 3; PRSS57, Serine protease 57; TNFAIP8, Tumor necrosis factor alpha-induced protein 8.

The exposure-outcome evidence landscape was curated and descriptively analyzed. Following data extraction and the exclusion of overlapping datasets based on sample size, a total of 496 exposure-outcome associations were identified, of which 346 were valid entries related to RA, covering 211 unique exposures. Of these exposures, 104 exposures demonstrated at least one significant causal association across 182 associations (Figure 4). The remaining 107 exposures, linked to 164 associations, yielded exclusively non-significant results (Supplementary Table S6).

Among the 104 exposures with significant findings, 58 were identified as potential risk factors. The most frequently reported risk factors included smoking behavior, body mass index (BMI), hypothyroidism, and certain inflammatory cytokines such as Interleukins-6 (IL-6) and IL-17. Conversely, 44 exposures were identified as protective factors, with educational attainment and cognitive function being the most prominent examples. Lastly, two exposures—sleep deprivation and sarcopenia—yielded conflicting results, being identified as both risk and protective factors in separate analyses.

### 3.6 Meta-analysis

**FIGURE 5:**
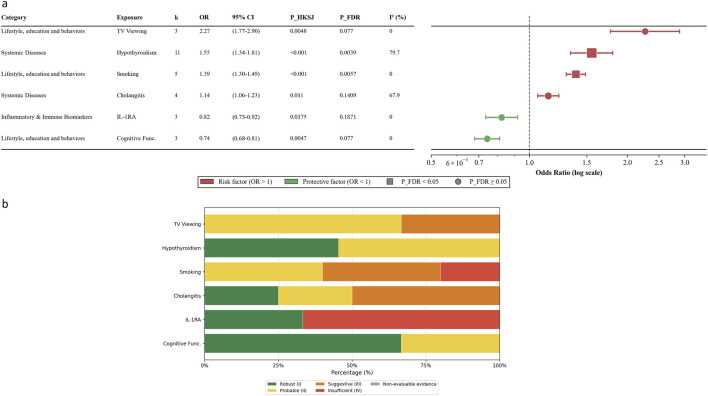
Causal associations and evidence strength grading of the six exposures associated with RA. **(a)** Forest plot. **(b)** Distribution of evidence strength grading.

A total of 200 exposure-RA associations across 65 unique exposures were reported more than twice, which were deemed eligible for quantitative synthesis. The meaningful results were summarized in Figure 5, and the full main analysis results were shown in Supplementary Table S7.

#### 3.6.1 Lifestyle, education and behaviors

Smoking was significantly associated with an increased risk of RA. The pooled estimate under the primary model was OR 1.39 (95% CI 1.30–1.49, P_HKSJ <0.001, P_FDR = 0.006), with low heterogeneity (I^2^ = 1.43%). This finding was consistent in sensitivity analyses. However, the evidence strength distribution was mixed, with 40% classified as Probable (II), 40% Suggestive (III), and 20% Insufficient (IV). Television viewing showed a nominally positive association with RA (OR 2.27, 95% CI 1.77–2.90, P_HKSJ = 0.005, P_FDR = 0.077), with evidence graded as Probable (II) (66.67%) and Suggestive (III) (33.33%). Similarly, cognitive function demonstrated a nominally protective effect (OR 0.74, 95% CI 0.68–0.81, P_HKSJ = 0.005, P_FDR = 0.077), supported by Robust (I) (66.67%) and Probable (II) (33.33%) evidence. Both associations were supported by sensitivity analyses using fixed-effect models (P < 0.001). No significant associations were observed for other lifestyle factors, including alcohol intake (P_FDR = 0.93), coffee intake (P_FDR = 0.38), or physical activity (P_FDR >0.05). Air pollution markers (NO2, NOx, PM10, PM2.5) did not show significant associations with RA in the primary analysis. The forest plots were shown in Supplementary Figure S1. The result of leave-one-out and sensitivity analyses were shown in Supplementary Tables S8, S9.

#### 3.6.2 Anthropometric

In the primary analysis using the HKSJ method, body mass index (BMI) was not significantly associated with RA (OR 1.32, 95% CI 0.96–1.81, P_HKSJ = 0.065, P_FDR = 0.38), despite showing significance in the fixed-effect sensitivity model (P < 0.001). High heterogeneity was observed for BMI (I^2^ = 78.9%). Waist-to-hip ratio (WHR) showed no association with RA (OR 1.00, 95% CI 0.47–2.13). The forest plots were shown in Supplementary Figure S2.

#### 3.6.3 Systemic diseases

Hypothyroidism was significantly associated with an increased risk of RA (OR 1.55, 95% CI 1.34–1.81, P_HKSJ <0.001, P_FDR = 0.004), with evidence graded as Robust (I) (45.45%) and Probable (II) (54.55%). Heterogeneity was substantial (I^2^ = 79.7%). Leave-one-out analysis confirmed the robustness of this finding, with no single study driving the effect. Due to enough studies (k = 11), further analyses were conducted. Subgroup analysis indicated that the association was significant in both the “Broad Definition” subgroup (OR 1.61, 95% CI 1.23–2.12) and the “Strict Definition” subgroup (OR 1.44, 95% CI 1.25–1.66) (Supplementary Table S3a; Supplementary Table S10). Univariate meta-regression showed that none of the examined covariates, including publication year, sample sizes, region, and SNP count, were significantly associated with the effect estimates (Supplementary Table S11). Publication bias was detected by Begg’s test (P = 0.041), although Egger’s test was non-significant (P = 0.346). The trim-and-fill method imputed six missing studies, resulting in a slightly attenuated but still significant adjusted OR of 1.48 (95% CI 1.33–1.66) (Supplementary Figure S3b). Cholangitis showed a nominally positive association (OR 1.14, 95% CI 1.06–1.23, P_HKSJ = 0.011, P_FDR = 0.14), with evidence graded as Robust (I) (25%), Probable (II) (25%) and Suggestive (III) (50%). No significant associations were found for inflammatory bowel disease, asthma, COVID-19, or other systemic diseases in the primary analysis. The forest plots were shown in Supplementary Figure S4.

#### 3.6.4 Inflammatory and immune biomarkers

IL-1 receptor antagonist (IL-1RA) showed a nominally inverse association with RA (OR 0.82, 95% CI 0.73–0.92, P_HKSJ = 0.018, P_FDR = 0.19). The evidence strength for IL-1RA was polarized, with 33.33% classified as Robust (I) but 66.67% as Insufficient (IV), suggesting that current data are not definitive. Other inflammatory biomarkers, including IL-6, IL-18, and TNF-α, did not demonstrate significant associations with RA in the primary analysis. The forest plots were shown in Supplementary Figure S5.

#### 3.6.5 Biomolecular and microbiomic factors

No significant associations were observed for minerals and trace elements (Iron, Copper, Magnesium, Zinc, Calcium) or sex hormone-binding globulin in the primary analysis (P_FDR >0.05 for all). The forest plots were shown in Supplementary Figure S6.

### 3.7 Associations with pRA and nRA based on single-study evidence

Our study analyzed 79 associations involving 67 exposures for pRA and 70 associations involving 59 exposures for nRA. For pRA, single-study evidence observed potential risk associations for smoking behavior, television viewing, basal metabolic rate (BMR), pulmonary fibrosis, IL-6, and Ruminococcaceae Ucg002. Conversely, computer usage, Alloprevotella, and Christensenellaceae R7 Group were observed to be potentially protective. Regarding nRA, preliminary findings indicated positive associations with coffee consumption, sleep deprivation, BMR, and inflammatory markers (IL-1RA, IL-18, IL-8), while IL-2R and several gut microbiota (*Actinomyces*, Lactobacillales, Terrisporobacter) showed protective estimates. Notably, BMR was observed as a risk factor in both subtypes, while Christensenellaceae R7 Group showed consistent protective estimates across both pRA and nRA. However, given that the majority of exposures associated with pRA and nRA were investigated in only a single MR study, meta-analytic synthesis was generally not feasible. Therefore, all findings should be suggested as hypothesis-generating rather than definitive causal conclusions. The landscape of these associations is illustrated in Supplementary Figure S7. Detailed characteristics for all identified associations were comprehensively listed in Supplementary Table S2.

## 4 Discussion

This systematic review and meta-analysis provided a comprehensive synthesis of the causal landscape of RA, integrating data from 117 Mendelian randomization studies that investigated 248 unique exposures. Our quantitative synthesis identified significantly causal evidence for hypothyroidism and smoking behavior were consistently associated with an increased risk of RA. Beyond the general RA outcome, our descriptive analysis indicated distinct etiological profiles for pRA and nRA subtypes. Despite the rapid acceleration of MR research in this field since 2022, our evidence grading suggests that while reporting quality is generally high, the strength of causal evidence remains variable, with only a small fraction of associations classified as robust, underscoring the need for continued rigorous investigation.

### 4.1 Lifestyle, education and behaviors

Our study demonstrated that smoking is significantly associated with an increased risk of RA. Numerous case-control studies and retrospective cohort studies have linked duration and intensity of smoking to an increased risk of RA (Hutchinson et al., 2001; Costenbader et al., 2006; Di Giuseppe et al., 2013; Di Giuseppe et al., 2014). A meta-analysis revealed that heavy smokers have a twofold increased risk of RA compared to non-smokers, with a clear dose-response relationship (Di Giuseppe et al., 2014). Moreover, smoking interacts with genetic risk factors such as the HLA-DRB1 shared epitope, significantly amplifying RA risk. For instance, in the Swedish EIRA cohort, smokers with shared epitope alleles had a tenfold increased risk of developing seropositive RA compared to those without these factors (Hedström et al., 2019). Smoking also interacts with other genetic variants, such as PADI4, further enhancing RA susceptibility (Kochi et al., 2011). These findings highlight the need for smoking prevention and cessation strategies to mitigate RA risk and improve overall health outcomes.

Prolonged sedentary behavior, especially during leisure activities such as watching television, has been shown in observational studies to have adverse effects on musculoskeletal health (Dzakpasu et al., 2021). Lack of physical activity is a significant contributor to chronic conditions, including musculoskeletal disorders (Booth et al., 2012). Our study supports a nominally significant association between the television watching and RA. Television viewing stands out as a predominant sedentary behavior due to its passive nature, leading to reduced energy expenditure and often increased caloric intake, particularly from snacking (Clark et al., 2011). This energy imbalance has been identified as a risk factor for weight gain, which is associated with conditions like low back pain and intervertebral disc degeneration.

Conversely, higher cognitive function was found to be nominally protective factors for RA. It aligns with studies. For example, Swedish patients with a university degree (compared with no university degree) had a 29% lower relative risk of developing RA (as opposed to the reported relative risk of 1.4) (Bengtsson et al., 2005); Those with ‘long term advanced studies’ (formal education >4 years) had lower odds of RA compared with those with no formal education (OR 0.30; 95% CI: 0.18, 0.51) (Pedersen et al., 2006). It suggesting that education may influence health behaviors and access to healthcare, thereby reducing RA risk.

### 4.2 Anthropometric

Although the primary analysis for BMI yielded insignificant association due to statistical strictness, the biological plausibility and sensitivity analyses suggest a potential link that warrants further investigation. Studies have shown that higher BMI and abdominal waist circumference are linked to an increased risk of RA. For example, indicate a stronger association between obesity and RA, particularly among women, compared to men (Ljung and Rantapää-Dahlqvist, 2016; Linauskas et al., 2019). Interestingly, the risk persists even when accounting for protective factors like physical activity, highlighting the intricate interplay of genetics and environmental factors in obesity and RA pathogenesis (Liu et al., 2019b). Mechanically, obesity promotes inflammation through multiple mechanisms. In obese individuals, macrophages are polarized toward pro-inflammatory (M1-like) states, which secrete cytokines such as TNF-α, IL-6, and IL-18, perpetuating chronic inflammation (Tang et al., 2009; Lumeng et al., 2007). This inflammatory environment also recruits CD4^+^ T-cells, resulting in a predominance of pro-inflammatory Th1 and Th17 cells while reducing anti-inflammatory Th2 and Treg cells (Liu and Nikolajczyk, 2019). Additionally, adipose tissue produces adipokines, such as leptin and adiponectin, that further contribute to RA-related inflammation (Liu and Nikolajczyk, 2019; Rodríguez et al., 2021). Elevated leptin levels in early RA are linked to increased reactive oxygen species, while adiponectin promotes bone erosion through osteoblast and osteoclast activation (Sun et al., 2017; Bemis et al., 2021). Other adipokines, including chemerin and resistin, have been associated with disease severity and clinical outcomes in RA (Maisha et al., 2023).

### 4.3 Systemic diseases

Our study identified hypothyroidism as a significantly associated risk factor for the development of RA, with the high quality of evidence graded. This aligns with existing literature that reports the prevalence of RA in patients with autoimmune thyroid disorders (AITD) is increased by 1–3-fold and *vice versa* the prevalence of AITD in patients with RA is increased by 1–6-fold (Weetman, 2011). In a large series of AITD patients from UK (n = 3,286), RA was the first autoimmune disorder associated to AITD, being present in 4.24% of Hashimoto thyroiditis ones. This strong epidemiological link suggests a shared immunological environment predisposing individuals to both conditions.

Regarding the substantial heterogeneity observed in the hypothyroidism-RA association, our analysis suggests a complex interplay of methodological and biological factors. While subgroup analysis indicated that phenotypic definitions contributed to this variation, biological mechanisms play a critical role. The shared genetic architecture between AITD and RA provides a key explanation. Included studies identified shared susceptibility loci, including the HLA region like HLA-DRB1 and non-HLA genes like TYK2, IL2RA, and PTPN22(85, 97). Furthermore, specific shared risk genes, including TYK2, IL2RA, and IRF5, are involved in cytokine signaling and T-cell activation common to both diseases (Peng et al., 2023). In MR analyses, these shared loci can introduce horizontal pleiotropy. Consequently, the inconsistency in how primary studies handled these variants-specifically whether they rigorously excluded the MHC region or pleiotropic SNPs-likely contributes to the residual heterogeneity. Thus, the observed association reflects both a direct causal effect and an underlying common autoimmune pathogenesis.

Cholangitis showed a nominally significant positive association with RA. This indicates a potential link between liver pathology and RA risk. The development of RA is often associated with liver dysfunction, which may result from concurrent autoimmune liver diseases (such as primary biliary cholangitis, PBC) or direct involvement of the liver parenchyma (Selmi et al., 2011). Studies indicate that PBC accounts for about 22% of patients with liver involvement in rheumatic conditions (Gebreselassie et al., 2019), supporting our finding of a potential causal connection.

### 4.4 Biological and inflammatory biomarkers

In terms of inflammatory biomarkers, our analysis yielded more conservative results compared to observational studies. We observed a nominally significant protective association between IL-1RA and RA risk. The evidence quality for IL-1RA was polarized, with a majority classified as Insufficient (IV), suggesting that while a potential link exists, the current genetic evidence is not definitive. Biologically, however, this potential protective effect is highly plausible. IL-1RA functions as a natural inhibitor of the pro-inflammatory cytokine IL-1, preventing it from binding to its receptor (Zhukova et al., 2025). This mechanism is clinically validated, as the recombinant human IL-1RA (Anakinra) is an approved therapeutic agent for reducing inflammation in RA patients (Kang et al., 2026). The alignment between our MR finding and established pharmacology supports the biological validity of IL-1RA as a protective factor, despite the statistical limitations.

### 4.5 Strengths, limitations, and future perspectives

This study provides a comprehensive systematic evaluation of RA risk factors using MR approaches. Its primary strengths lie in the extensive scope of exposures covered and the rigorous meta-analysis, which effectively mitigates the biases inherent in traditional observational studies.

However, several limitations must be acknowledged. First, the predominance of data from European populations restricts the generalizability of our findings to other ethnicities. Second, to ensure methodological homogeneity, we restricted our inclusion to two-sample MR. While this maximized the use of large-scale summary statistics, it excluded insights derived from one-sample MR, multivariable MR, and non-linear MR designs. Third, the substantial proportion of evidence classified as non-evaluable or insufficient highlights the challenges posed by data heterogeneity and the inherent assumptions of genetic instruments.

Future research should prioritize improving data quality in underrepresented populations and exposure categories. Furthermore, integrating advanced designs, such as multivariable MR, will be essential to validate these findings and elucidate the biological mechanisms linking identified risk factors to RA pathogenesis.

## 5 Conclusion

This systematic review and meta-analysis refine the etiological landscape of rheumatoid arthritis, distinguishing robust causal drivers from tentative associations through rigorous methodological standards. The definitive identification of smoking behavior and hypothyroidism as key risk factors, alongside the protective role of cognitive function, provides a clear evidence base for prioritizing lifestyle interventions and comorbidity management in clinical practice. Furthermore, our application of conservative statistical adjustments reveals the uncertainty surrounding other commonly cited risk factors, cautioning against the premature translation of insufficient evidence. Future research must now pivot from broad screening to high-quality, subtype-specific investigations to fully unravel the complex, multifactorial pathogenesis of RA.

## Data Availability

The original contributions presented in the study are included in the article/Supplementary Material, further inquiries can be directed to the corresponding authors.
